# Supplementary material for: Intrinsically stretchable organic photovoltaics by redistributing strain to PEDOT:PSS with enhanced stretchability and interfacial adhesion
Source: Nat Commun. 2024 Jun 8;15:4902. doi: 10.1038/s41467-024-49352-4 (PMC11162488; doi:10.1038/s41467-024-49352-4)
Supplement: Supplementary file 1 — Supplementary Information [file 41467_2024_49352_MOESM1_ESM.pdf]

***Supplementary Information***

# Intrinsically Stretchable Organic Photovoltaics by Redistributing Strain to PEDOT:PSS with Enhanced Stretchability and Interfacial Adhesion

*Jiachen Wang,[1,2,3] Yuto Ochiai,[2] Niannian Wu,[2,4] Kiyohiro Adachi,[2] Daishi Inoue,[2] Daisuke Hashizume,[2] Desheng Kong,[5] Naoji Matsuhisa,[6,7] Tomoyuki Yokota,[1,8] Qiang Wu,[9] Wei Ma,[9] Lulu Sun,[3] Sixing Xiong,[2] Baocai Du,[1,2] Wenqing Wang [1,2] Chih-Jen Shih,[3] Keisuke Tajima,[2] Takuzo Aida[2,4], Kenjiro Fukuda,*[2,10] and Takao Someya*[1,2,10]*

[1]Department of Electrical Engineering and Information Systems, The University of Tokyo, 7-3-1 Hongo, Bunkyo-ku, Tokyo 113-8656, Japan

[2]RIKEN Centre for Emergent Matter Science (CEMS), 2-1 Hirosawa, Wako, Saitama 351-0198, Japan

[3]Institute for Chemical and Bioengineering, ETH Zurich, Zurich 8093, Switzerland

[4]Department of Chemistry and Biotechnology, School of Engineering, The University of Tokyo, 7-3-1 Hongo, Bunkyo-ku, Tokyo 113-8656, Japan

[5]College of Engineering and Applied Sciences, State Key Laboratory of Analytical Chemistry for Life Science, Nanjing University, Nanjing 210046, China

[6]Research Center for Advanced Science and Technology, The University of Tokyo, 4-6-1 Komaba, Meguro-ku, Tokyo 153-8505, Japan

[7]Institute of Industrial Science, The University of Tokyo, 4-6-1 Komaba, Meguro-ku, Tokyo 153-8505, Japan

[8]Institute of Engineering Innovation, School of Engineering, The University of Tokyo, 7-3-1 Hongo, Bunkyo-ku, Tokyo 113-8656, Japan

[9]State Key Laboratory for Mechanical Behaviour of Materials, Xi'an Jiaotong University, Xi'an 710049, P. R. China

[10]Thin-Film Device Laboratory, RIKEN, 2-1 Hirosawa, Wako, Saitama 351-0198, Japan

**Supplementary Table 1.** Sheet resistance and transparency of conductive PEDOT:PSS films with EG and 0, 2, 5, and 10 mg mL$^{-1}$ ION E additive spin-coated at 2000 rpm.

| | **Film thickness (nm)** | **R□ (Ω/□)** | **Transparency at 550 nm (%)** |
|---|---|---|---|
| Conductive PEDOT:PSS | 60 | 202 | 87.8 |
| Conductive PEDOT:PSS with 2-ION E | 69 | 203 | 88.1 |
| Conductive PEDOT:PSS with 5-ION E | 98 | 206 | 88.3 |
| Conductive PEDOT:PSS with 10-ION E | 113 | 205 | 88.0 |

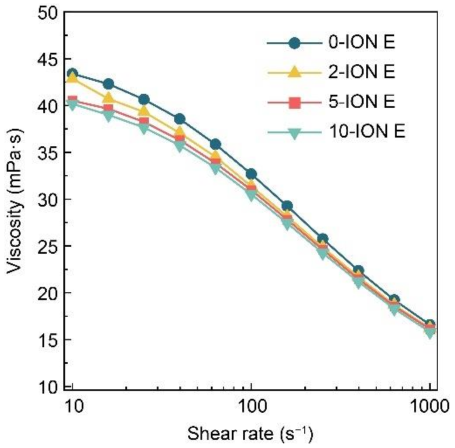


**Supplementary Fig. 1.** The dependency of dynamic viscosity on shear rate for conductive PEDOT:PSS solutions with different amounts of ION E additive.

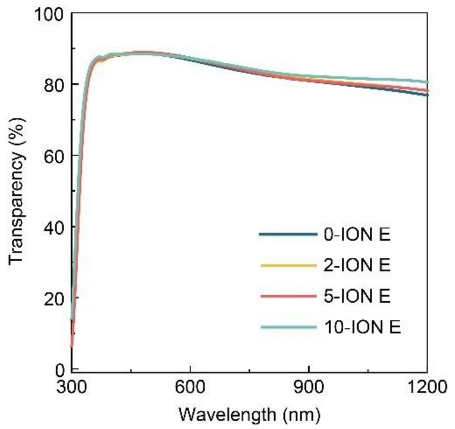


**Supplementary Fig. 2.** Transparency of conductive PEDOT:PSS films with different amounts of ION E additive.

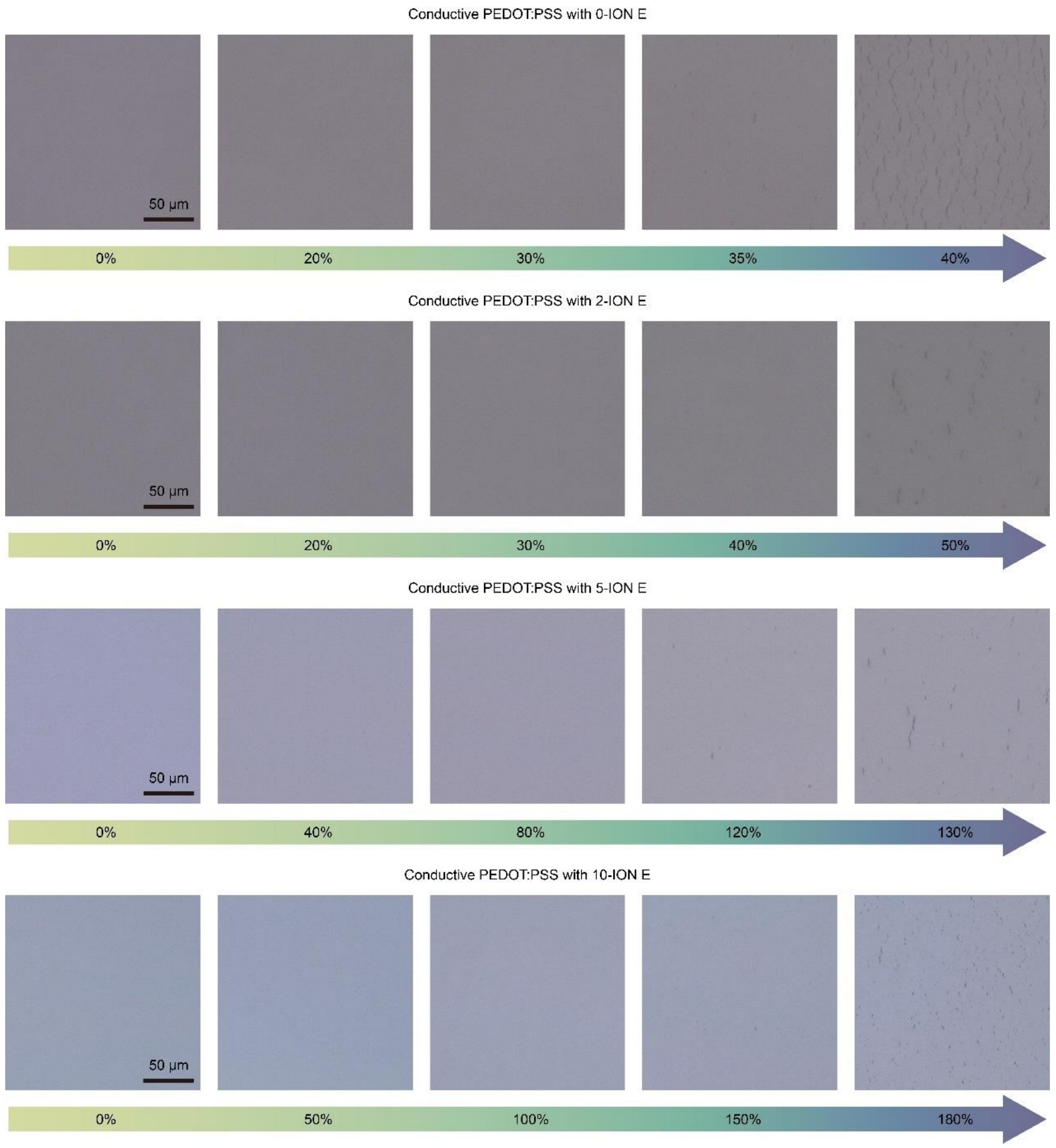


**Supplementary Fig. 3.** Optical microscopy (OM) images of PU//conductive PEDOT:PSS films with 0, 2, 5, and 10 mg $mL^{-1}$ ION E additive subjected to different tensile strains.

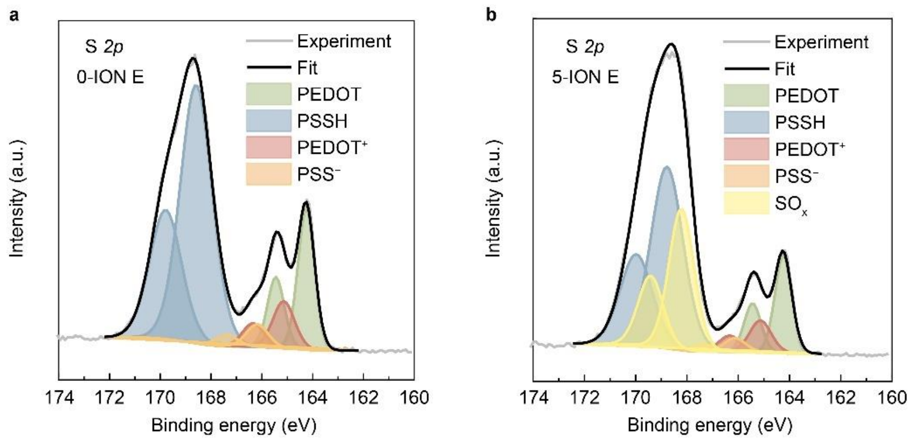


**Supplementary Fig. 4.** S (*2p*) XPS profiles of the surfaces of conductive PEDOT:PSS a) without ION E and b) with 5-ION E.

**Supplementary Note 1. XPS Analysis of Conductive PEDOT:PSS without and with 5-ION E**

XPS analysis was conducted to confirm the elemental states in the conductive PEDOT:PSS films with and without the ION E additive. The S (*2p*) signals shown in Supplementary Fig. 4 can be attributed to four distinct components originating from unique chemical environments: pristine PEDOT, doped $PEDOT^{+}$, PSS-H, and $PSS^{-}$.[3] The peaks between 172 and 166 eV correspond to the sulfonate groups in PSS, whereas those within the 166–163 eV range can be ascribed to the sulphur atoms in PEDOT.[4] Additionally, the new peaks of $SO_x$ representing ION E (Supplementary Fig. 5) can be observed in Supplementary Fig. 4b. The PSS–PEDOT area ratios for the conductive PEDOT:PSS systems without and with 5-ION E were calculated to be 2.58:1 and 2.61:1, respectively, indicating that ION E did not introduce new covalent bonds.

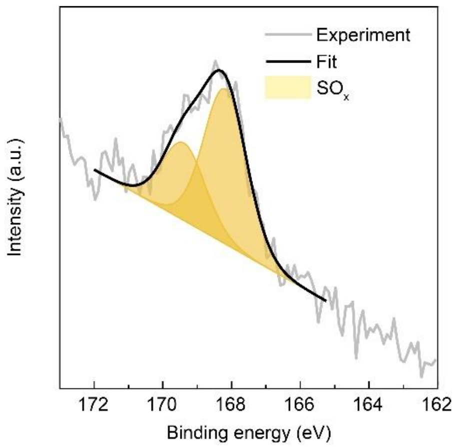


**Supplementary Fig. 5.** S (*2p*) XPS profile of ION E.

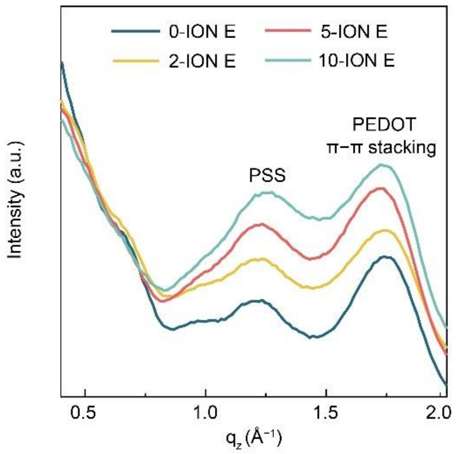


**Supplementary Fig. 6.** Out-of-plane 1D plot of conductive PEDOT:PSS films with different amounts of ION E additive, extracted from the cake cuts of the 2D GIWAXS patterns with $\beta$ range of 160° to 170° shown in Supplementary Fig. 6.

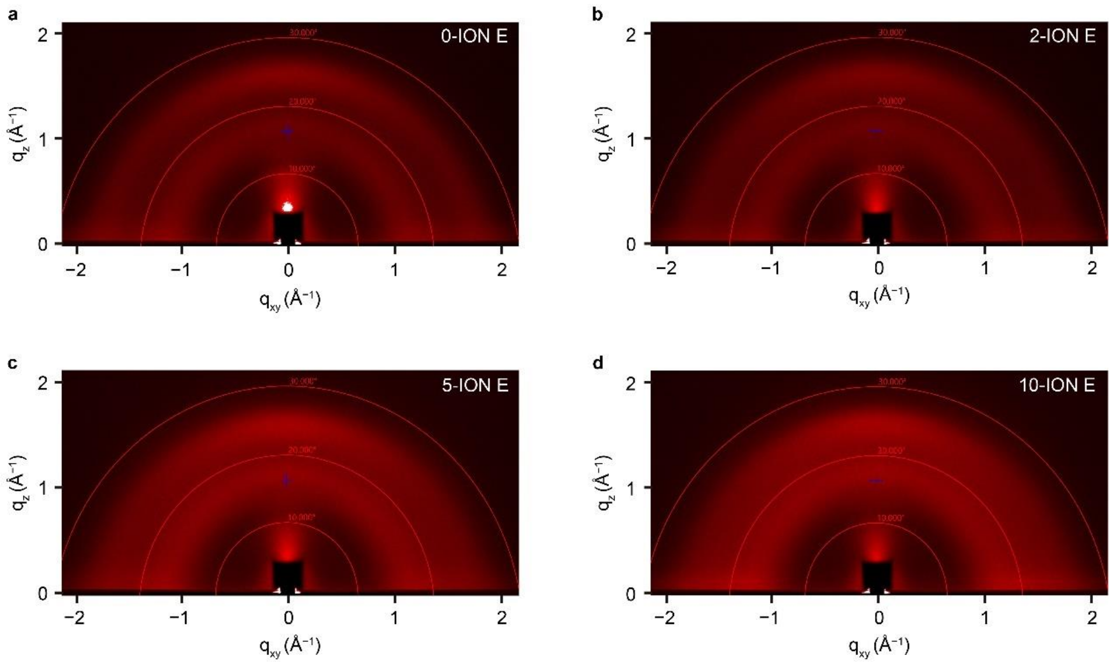


**Supplementary Fig. 7.** 2D GIWAXS patterns of conductive PEDOT:PSS films a) without ION E and with b) 2 mg $mL^{-1}$, c) 5 mg $mL^{-1}$, and d) 10 mg $mL^{-1}$ ION E.

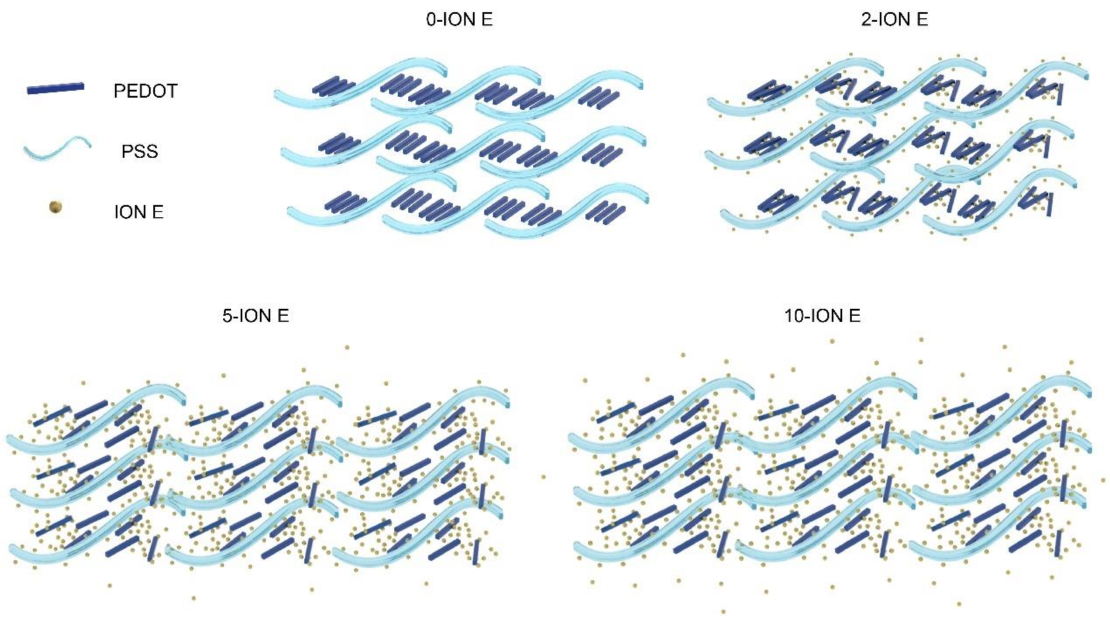


**Supplementary Fig. 8.** Schematic images of the crystalline structures of conductive PEDOT:PSS with different concentrations of ION E.

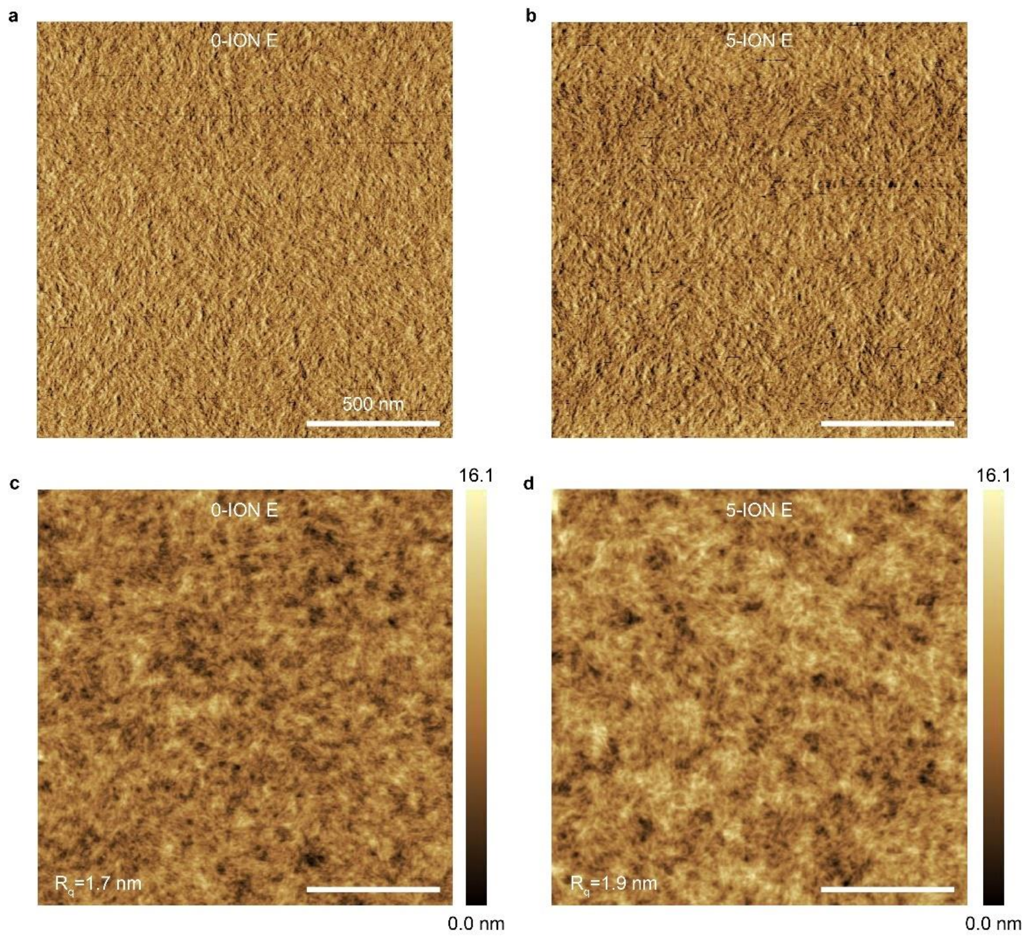


**Supplementary Fig. 9.** AFM-based phase images of conductive PEDOT:PSS films a) without ION E and b) with 5 mg mL$^{-1}$ ION E. AFM-derived morphology images of conductive PEDOT:PSS films c) without ION E and d) with 5 mg mL$^{-1}$ ION E.

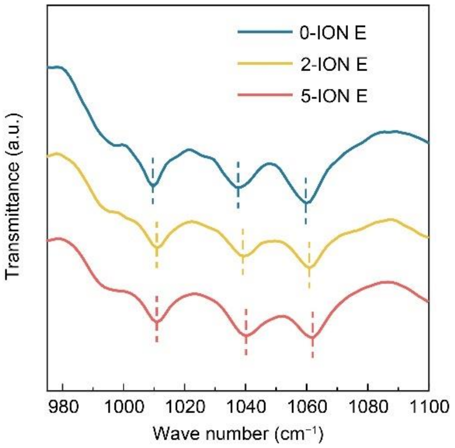


**Supplementary Fig. 10.** FTIR spectra of conductive PEDOT:PSS films with different ION E concentrations.

**Supplementary Note 2 Interactions between ION E and PEDOT:PSS**

The interactions between ION E and PEDOT:PSS were revealed by FTIR spectroscopy. The FTIR bands in the ranges of 1005−1010 $cm^{-1}$ and 1035−1040 $cm^{-1}$ featured the stretching vibrations of the sulfonic group ($SO_3H$) of PSSH.[5] Both peaks shifted to larger wave numbers with higher concentrations of ION E, demonstrating that the FTIR bands blue-shifted, indicating a reduction in hydrogen bonds among the PSSH.[6] Moreover, the C—O—C stretching band of PEDOT appeared at 1059 $cm^{-1}$ in the absence of 0-ION E and shifted to 1062 $cm^{-1}$ after the incorporation of 5-ION E, which is also indicative of the interaction between PEDOT:PSS and ION E.[7]

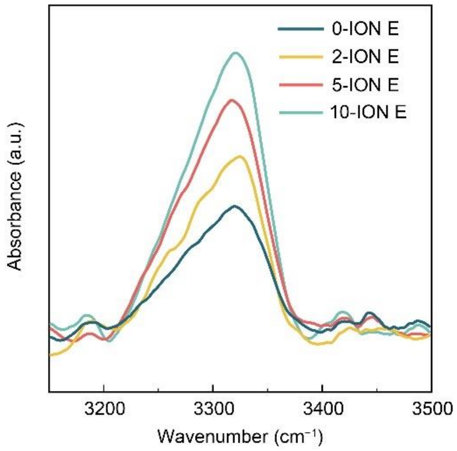


**Supplementary Fig. 11.** Attenuated total reflectance-Fourier transform infrared spectra of conductive PEDOT:PSS with different amounts of ION E on PU film.

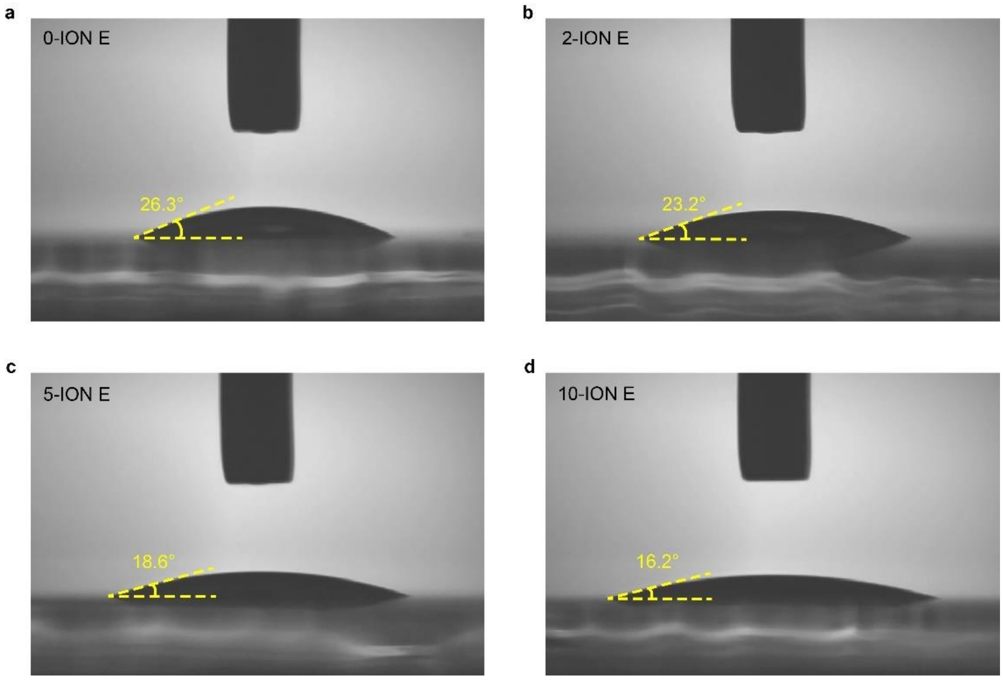


**Supplementary Fig. 12.** Contact angles of conductive PEDOT:PSS droplets with a) 0-ION E, b) 2-ION E, c) 5-ION E and d) 10-ION E placed on a PU surface.

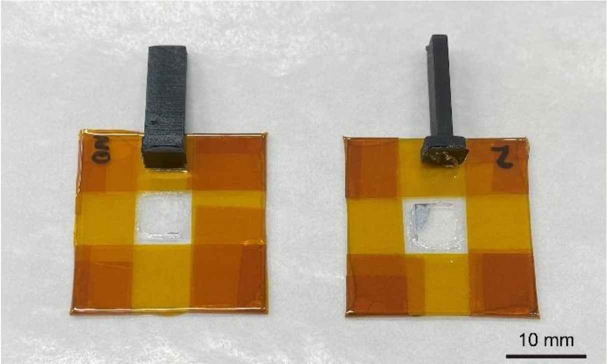

**Supplementary Fig. 13.** Photographs of samples after pull-off tests.

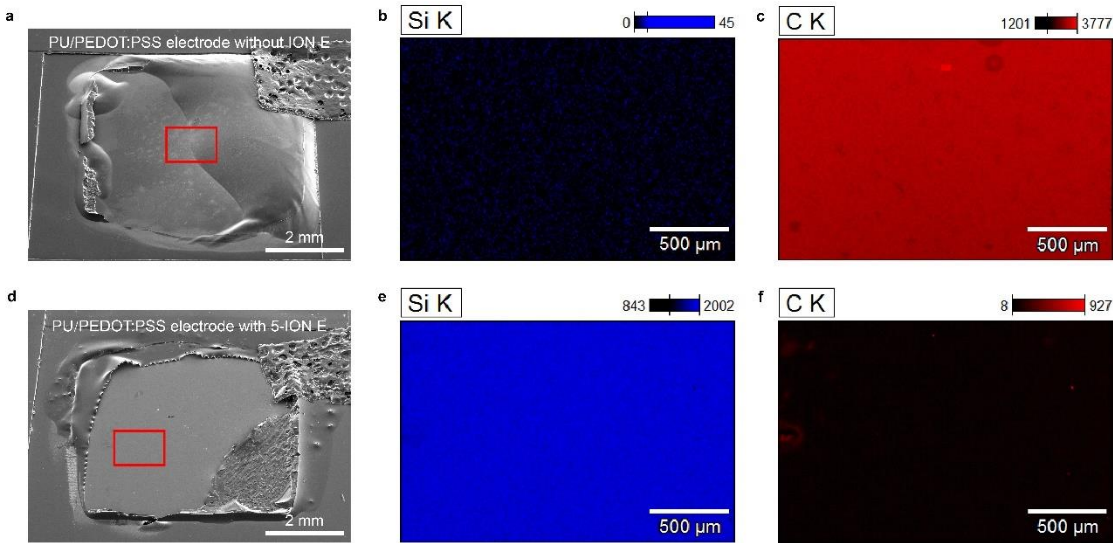


**Supplementary Fig. 14.** a, d) Tilted (45°) SEM images of the exposed surface at the substrate side after pull-off tests. EDX maps of b, e) Si and c, f) C. The EDX mapping areas are marked by red boxes in Panels (a) and (d).

**Supplementary Note 3. SEM–EDX Mapping of Exposed Surface at the Substrate Side**

EDX maps of Si and C clearly revealed an intense C signal but no Si signals in the absence of ION E, indicating the existence of a residual carbon-based PU layer on the surface. This implies that the adhesion between PU and glass was significantly stronger than that between PU and pristine conductive PEDOT:PSS. Therefore, delamination occurred at the interface between the conductive PEDOT:PSS and the PU substrate. In sharp contrast, the sample with 5-ION E showed an intense Si signal but almost no C signal, indicating the exposure of the bottom glass substrate. This demonstrated that the adhesion between PU and conductive PEDOT:PSS with 5-ION E was superior to that between PU and glass, resulting in delamination at the interface between PU and the bottom glass substrate. The SEM results confirmed a noteworthy enhancement in the interfacial adhesion between the conductive PEDOT:PSS films with ION E and the PU substrate.

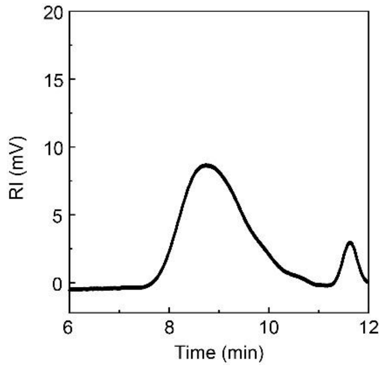


**Supplementary Fig. 15.** Gel permeation chromatography (GPC) conducted with a RI detector for Ter-D18.

**Supplementary Table 2.** $M_n$, $M_w$, and $Mw/Mn$ values of Ter-D18, as obtained from the GPC-RI measurements.

| GPC-RI | | |
|---|---|---|
| $M_n$ | $M_w$ | $M_w/M_n$ |
| 89,760 | 339,651 | 3.78 |

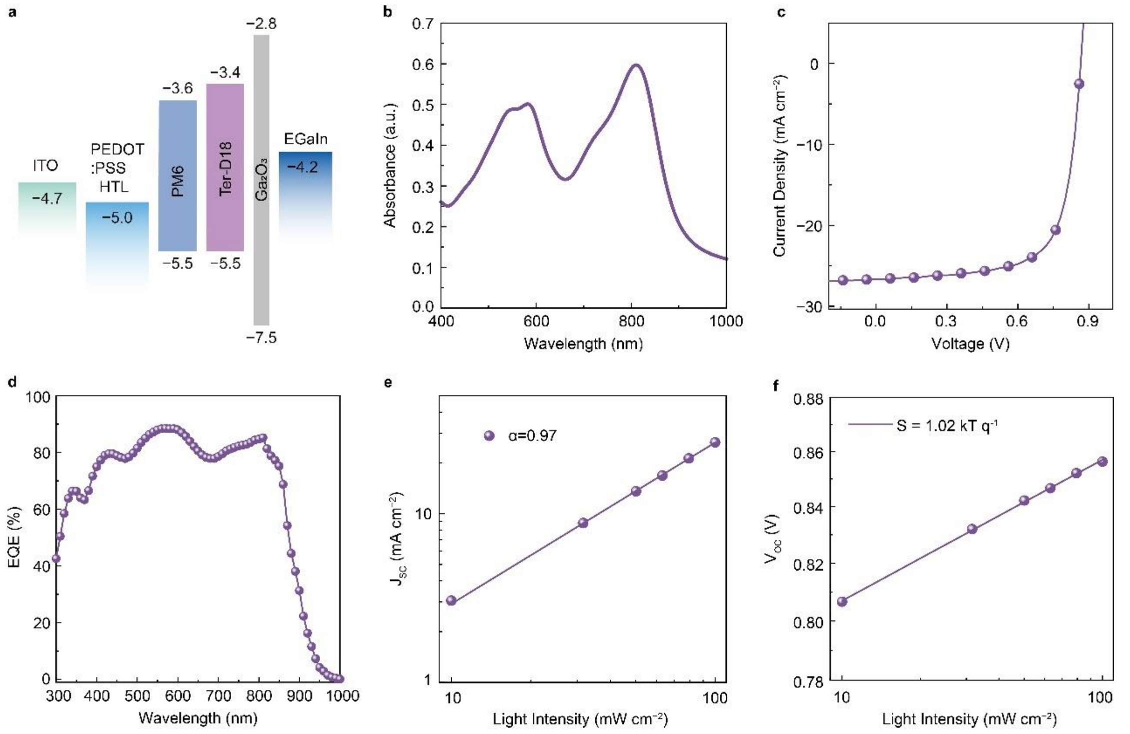


**Supplementary Fig. 16.** Performance of OPV device with the Ter-D18:Y6 active layer. a) Energy-level diagram of OPV device assembled as follows: ITO//PEDOT:PSS HTL//Ter-D18:Y6//EGaIn. b) Absorption spectrum of 98-nm-thick Ter-D18:Y6 active layer. c) *J*–*V* curves acquired under AM 1.5G illumination at 100 mW cm$^{-2}$. d) spectrum. Dependence of e) short-circuit current and f) open-circuit voltage on light intensity.

## Supplementary Note 4. Performance of OPV Device with Ter-D18:Y6 Active Layer

Supplementary Fig. 16a shows the energy-level diagram of the ETL-free OPV device with an EGaIn cathode, and Supplementary Fig. 16b shows the absorption spectrum of a 98-nm-thick Ter-D18:Y6 active layer. The newly developed active system showed excellent performance with an average short-circuit current density ($J_{SC}$) of 26.67 mA cm$^{-2}$, open-circuit voltage ($V_{OC}$) of 0.87 V, fill factor (FF) of 0.70, and PCE of 16.19% (Supplementary Fig. 16c). The $J_{SC}$ value estimated from the EQE spectra (26.87 mA cm$^{-2}$; Supplementary Fig. 16d) is consistent with the $J_{SC}$ value derived from the *J*–*V* plots. Additionally, charge recombination was qualitatively investigated by measuring the light intensity dependence of $J_{SC}$ and $V_{OC}$. As shown in Supplementary Fig. 16e, the device exhibited a near-linear power-law dependence of $J_{SC}$ on light intensity ($J_{SC} \propto I_{\alpha}$)[8] with an α value of 0.97, indicating only a minor contribution of the bimolecular recombination under short-circuit conditions in the aforementioned device structure. Furthermore, the slope of $V_{OC}$ against light intensity is 1.02 kT/q (Supplementary Fig. 16f), suggesting negligible charge-trapping effects.

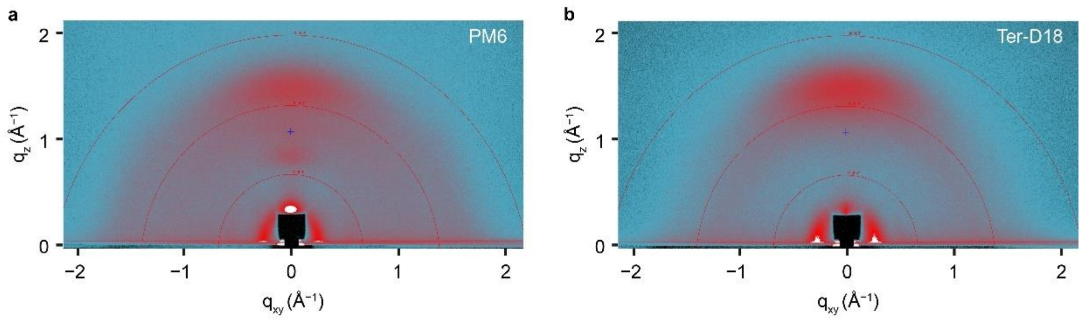


**Supplementary Fig. 17.** 2D GIWAXS patterns of a) PM6 and b) Ter-D18 films.

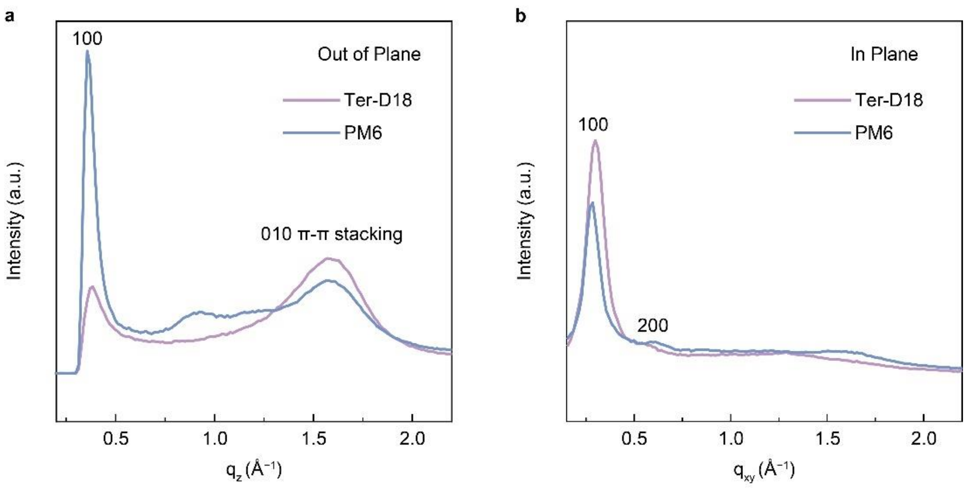


**Supplementary Fig. 18.** line-cut profiles in the a) pseudo-out-of-plane (OOP) and b) in-plane (IP) directions.

**Supplementary Note 5. 2D GIWAXS analysis of Ter-D18 and PM6 films.**

The 1D line-cut profiles are extracted at different azimuth angles $\beta$, which is defined as the angle between the $q$ vector and $q_z$ (from 90° to 270° in our system). The 1D in plane (IP) line-cut profile is obtained by calculating the integrated profile from the 2D GIWAXS frame with a polar integration of $\beta$ from 95° to 105° for the observed $q$ range. The 1D out of plane (OOP) line-cut profile is obtained by calculating the integrated profile from the 2D GIWAXS frame with a polar integration of $\beta$ from 160° to 170° for the observed $q$ range.[9, 10]

From 2D GIWAXS patterns of neat films, it is evident that both Ter-D18 and PM6 display a dominant face-on molecular orientation. PM6 shows strong 100 and weak 200 diffraction peaks at 0.28 and 0.61 $Å^{-1}$ in the IP direction,[11] whereas the 100 peak shifted to a larger $q_{xy}$ for Ter-D18, which suggests a reduced *d*-spacing. The correspondingly enhanced full width at half maximum (FWHM) of 100 peak from 0.085 $Å^{-1}$ for PM6 to 0.100 $Å^{-1}$ for Ter-D18 further demonstrated a smaller crystal grain size of Ter-D18 based on Scherrer equation.[12] Additionally, in contrast to Ter-D18 that demonstrated only one single primary diffraction peak when $q_z$ is larger than 0.5 $Å^{-1}$ in the pseudo-OOP direction, PM6 exhibited multiple diffraction peaks at 0.35, 0.92 and 1.57 $Å^{-1}$, indicating the stronger crystallinity. Moreover, both the Ter-D18 and PM6 neat films exhibited a typical 010 peak, which is assigned to the π–π stacking, in the OOP direction located at $q_z = 1.57$ $Å^{-1}$, corresponding to the same *d*-spacing value of 4.00 Å. Similarly, the correspondingly wider FWHM of 0.321 $Å^{-1}$ for Ter-D18 than 0.221 $Å^{-1}$ for PM6 demonstrated a smaller crystal grain size of Ter-D18 compared to PM6. The above results show that Ter-D18 possesses a weakened crystallinity while enables the formation of a face-on molecular orientation, promoting effective exciton separation and carrier transport.[13]

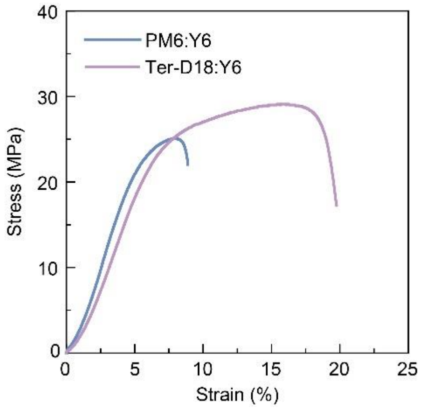


**Figure S19.** Stress-strain curves of the pseudo-free-standing PM6:Y6 and Ter-D18:Y6 active films.

**Supplementary Table 3.** Crack-onset strain and toughness of PM6:Y6 and Ter-D18:Y6 active films.

| Active system | Crack-onset strain (COS) | Toughness (MJ $m^{-3}$) |
|---|---|---|
| PM6:Y6 | 8.14 | 1.43 |
| Ter-D18:Y6 | 14.81 | 4.30 |

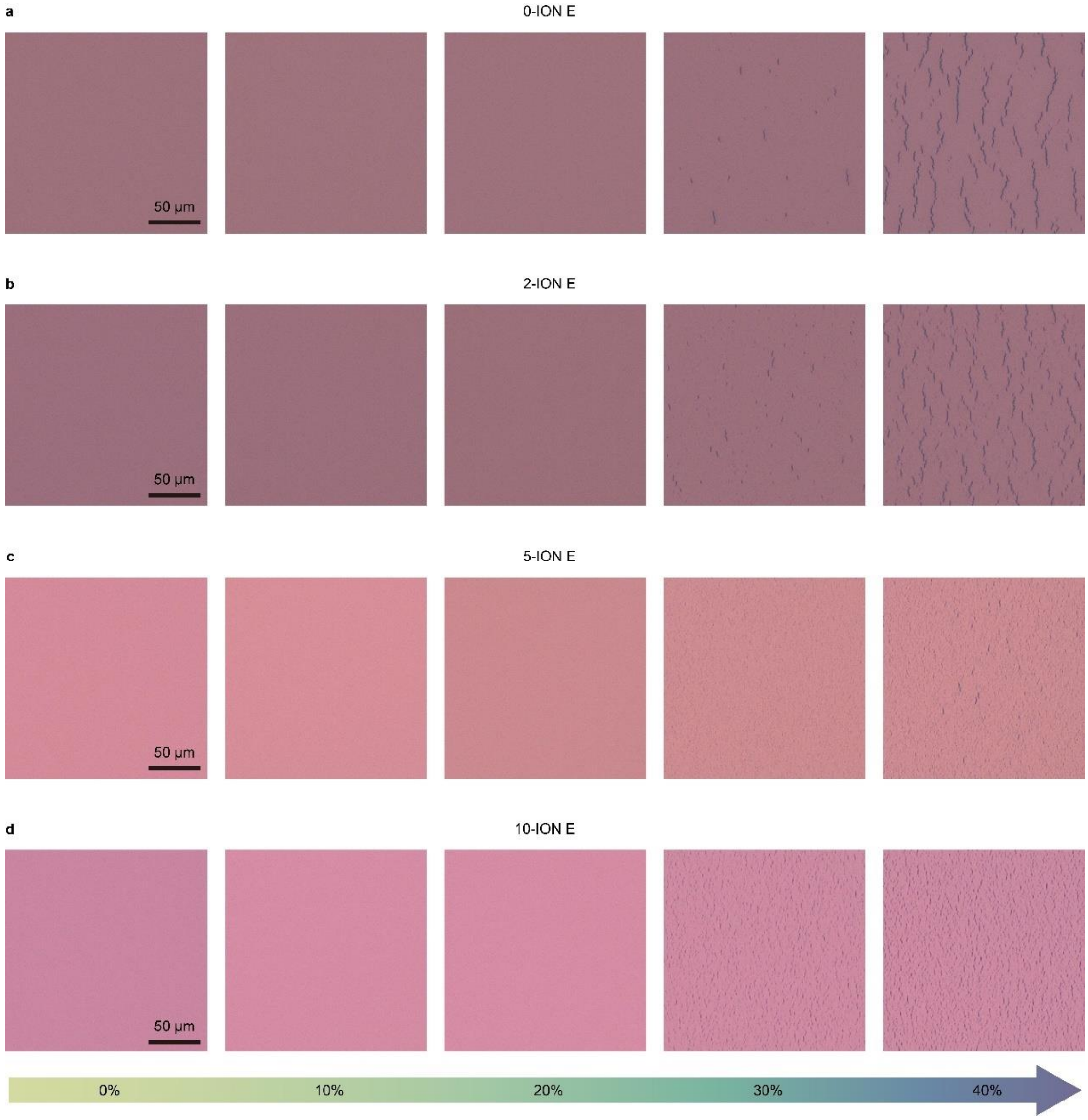


**Supplementary Fig. 20.** OM images of freestanding PU//conductive PEDOT:PSS with ION E//PM6:Y6 films containing a) 0-ION E, b) 2-ION E, c) 5-ION E, and d) 10-ION under 0%, 10%, 20%, 30%, and 40% strains.

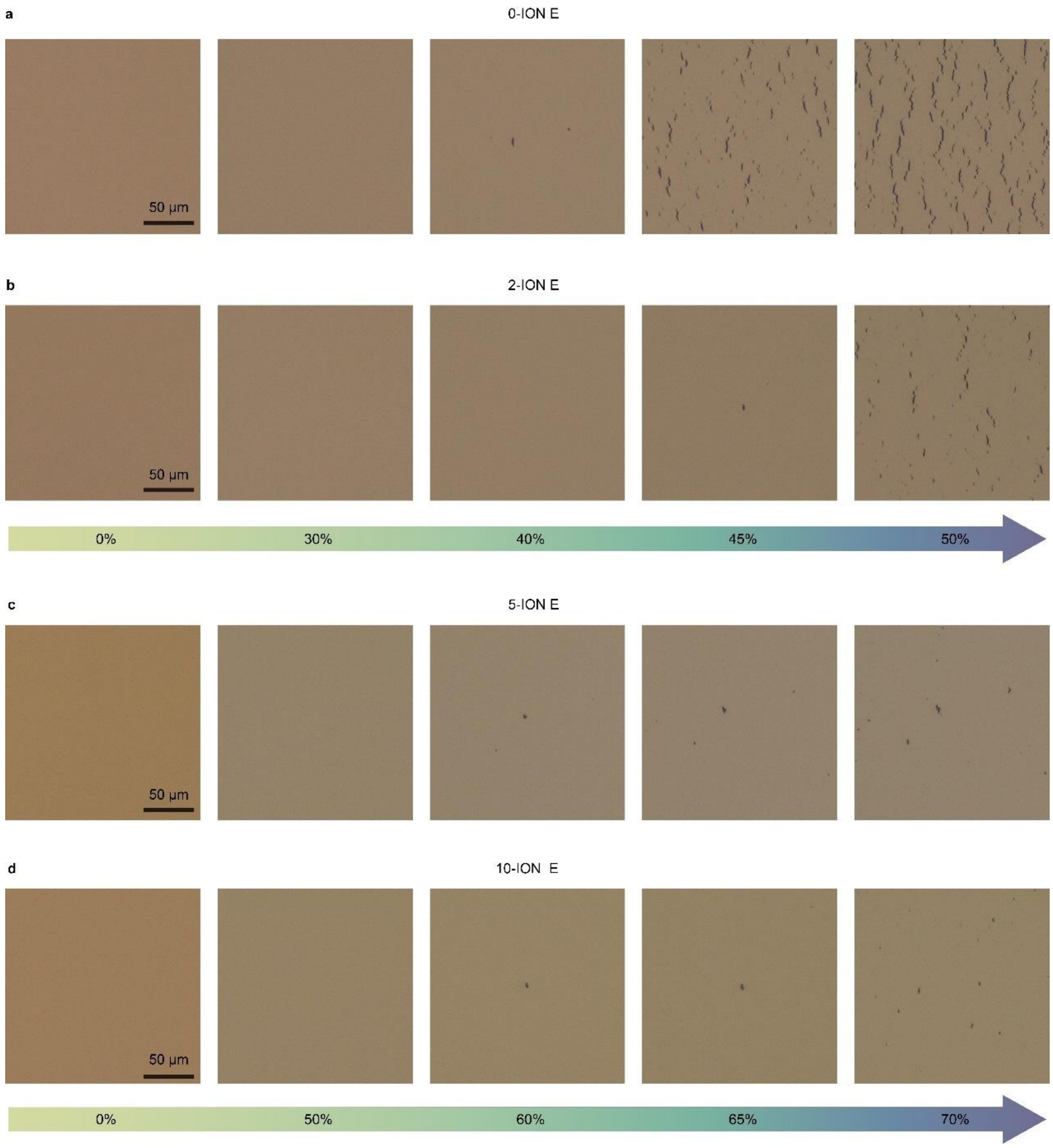


**Supplementary Fig. 21.** OM images of freestanding PU//conductive PEDOT:PSS with ION E//Ter-D18:Y6 films containing a) 0-ION E and b) 2-ION E under 0%, 30%, 40%, 45%, and 50% strains. OM images of similar films containing c) 5-ION E and d) 10-ION E under 0%, 50%, 60%, 65%, and 70% strains.

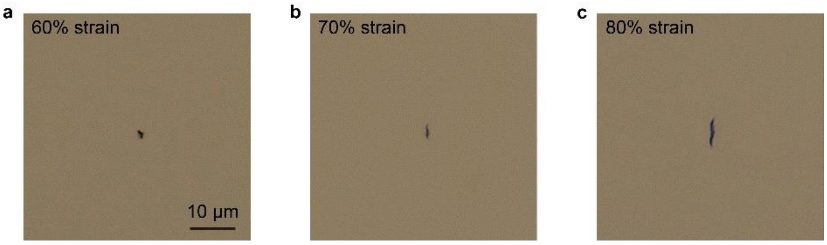


**Supplementary Fig. 22.** High-magnification OM images of freestanding PU//conductive PEDOT:PSS with 5-ION E//Ter-D18:Y6 films under a) 60%, b) 70% and c) 80% strains.

**Supplementary Note 6. OM-Imaged Surface Morphology of Freestanding PU//conductive PEDOT:PSS/active layer with Varying Amounts of ION E and Different Active Layers**

Cracks appeared at 30% strain in all the composite films containing varying concentrations of ION E and the PM6:Y6 active layer (Fig. 4c and Supplementary Fig. 19). Notably, in the films containing 0-ION E and 2-ION E, the initiated cracks were considerably larger and more apparent than those in the films with 5-ION E and 10-ION E, with the surfaces of the latter exhibiting minuscule densely distributed cracks. Additionally, crack propagation was considerably suppressed in the samples with 5-ION E and 10-ION E, which showed intermittent, short cracks under severe tensile strains of up to 40%. In contrast, crack generation was delayed in the composite films with the Ter-D18:Y6 active layer, given that the COS values increased gradually from 40% in the absence of ION E to 45% in the presence of 2-ION E. Impressively, the composite films with 5-ION E and 10-ION E even withstood a tensile strain of 60% without generating any cracks (Fig. 4f and Supplementary Fig. 20). Additionally, the propagated cracks in the composite films with the Ter-D18:Y6 active layer were significantly smaller and scattered than those in the films with PM6:Y6 even under considerably harsher tensile strains of up to 70%, demonstrating the superior mechanical durability of Ter-D18:Y6 active system in comparison to the PM6:Y6 counterpart. Additionally, there were no sub-micro cracks around the visible cracks observed at higher microscope magnifications upon reaching the COS (Supplementary Figs. 21).

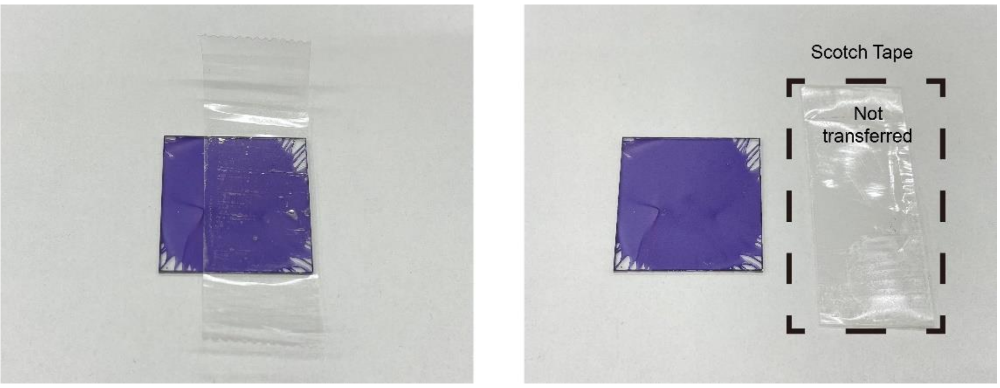


**Supplementary Fig. 23.** Optical images of the scotch tape test. The active layer cannot be transferred to scotch tape, demonstrating robust bonding between the active layer and the underlying PEDOT:PSS layers.

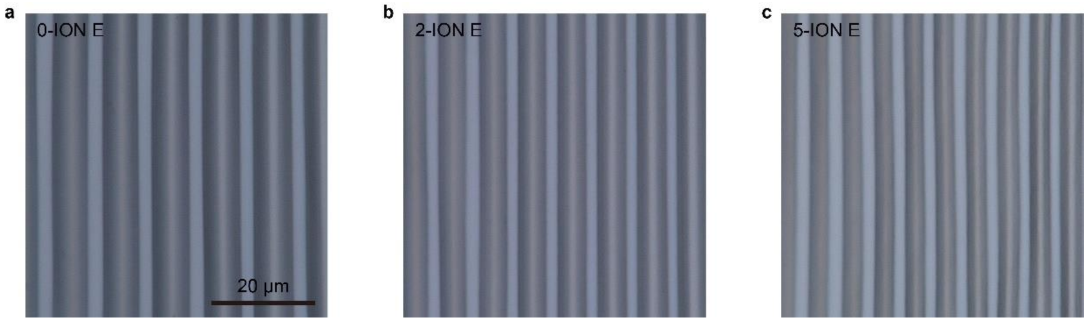


**Supplementary Fig. 24.** Representative OM images of buckled conductive PEDOT:PSS films with a) 0-ION E, b) 2-ION E and c) 5-ION E with a film thickness of ~100 nm on PDMS substrate.

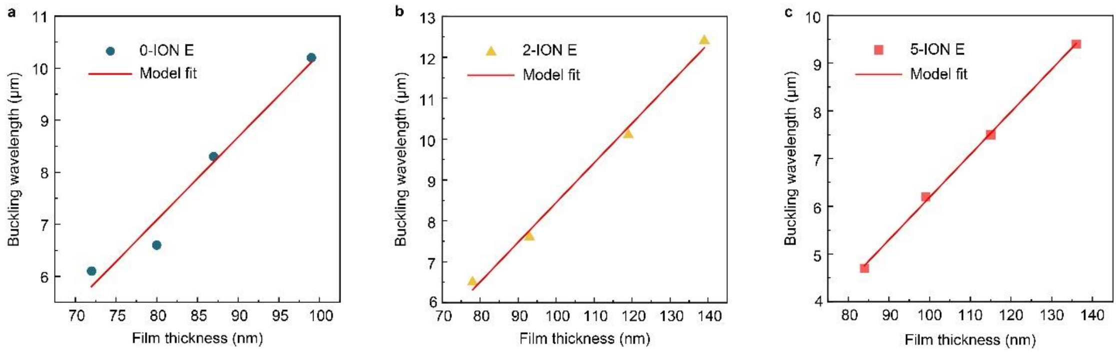


**Supplementary Fig. 25.** Experimental data of buckling wavelength of conductive PEDOT:PSS films with a) 0-ION E, b) 2-ION E and c) 5-ION as a function of film thickness.

In buckling-based metrology, a thin film of material is deposited on a soft, compliant elastomeric substrate such as polydimethylsiloxane (PDMS). Under compressive strain, the film buckles, creating a wavy, wrinkled surface. By applying the well-known buckling formulas in conjunction with the measured buckling wavelength and other relevant material properties, the mechanical modulus of the film material can be extracted.[2]

The mechanical modulus of film materials can be calculated according to the following equation:

$$d = 2\pi h \left[ \frac{(1 - v_s^2) E_f}{(1 - v_f^2) E_s} \right]^{1/3}$$

where d and h represent the buckling wavelength and the thickness of the upper film, respectively. $v_s$ and $v_f$ represent the Poisson's ratio of the PDMS substrate (0.5) and the film material (0.35), respectively. $E_s$ and $E_f$ are the Young's modulus of the PDMS substrate and the film material.

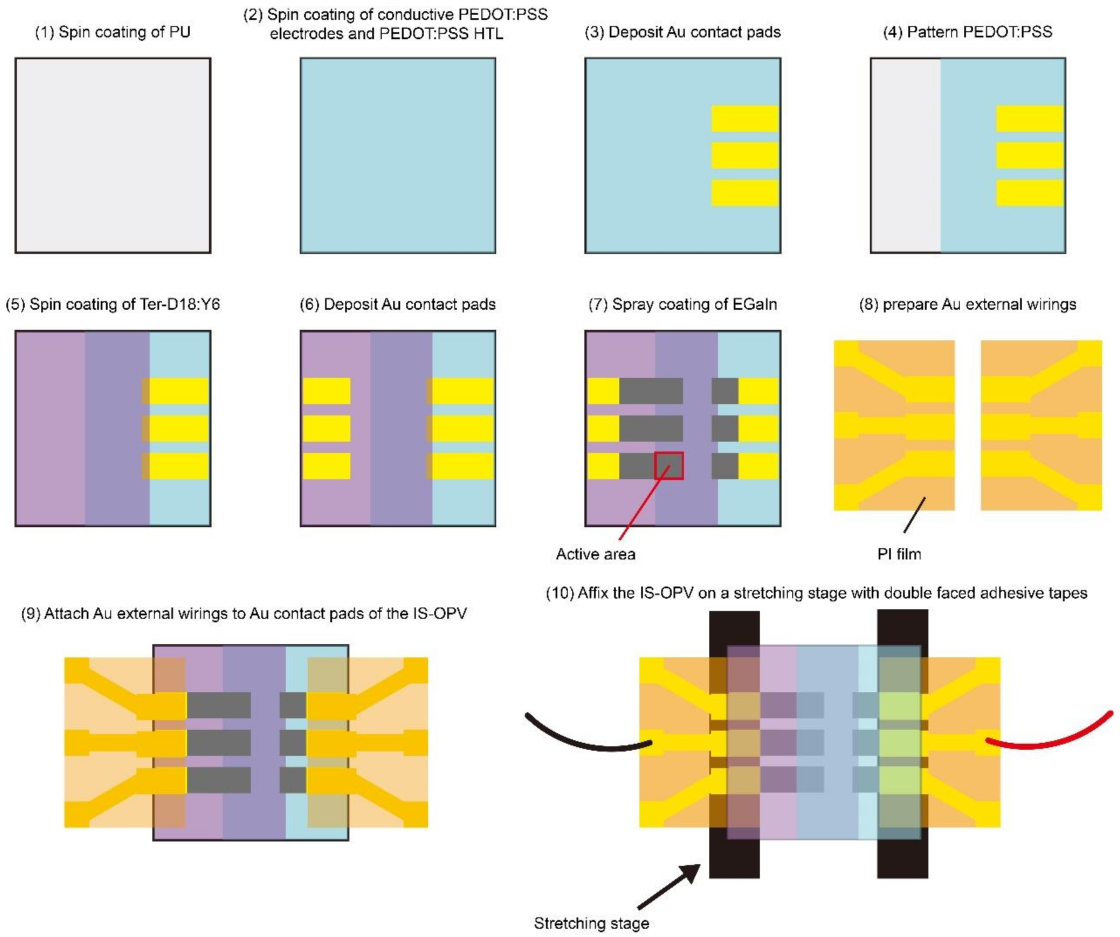


**Supplementary Fig. 26.** Schematic of the fabrication process of IS-OPVs. (1) Spin coating of PU substrate on an OTS-modified glass. (2) Spin coating of conductive PEDOT:PSS electrodes and PEDOT:PSS hole transport layer. (3) Deposit Au contact pads for bottom electrodes. (4) Pattern PEDOT:PSS via oxygen plasma treatment. (5) Spin coating of Ter-D18:Y6 active layer and wipe the edge of Au contact pads. (6) Deposit Au contact pads for top electrodes. (7) Spray coating of EGaIn with a shadow mask. (8) Prepare Au external wirings. (9) Attach Au external wirings to the edge of Au contact pads of the IS-OPVs using an electrically conductive adhesive-transfer tape. (10) Affix the IS-OPV on a stretching stage with double-faced adhesive tapes.

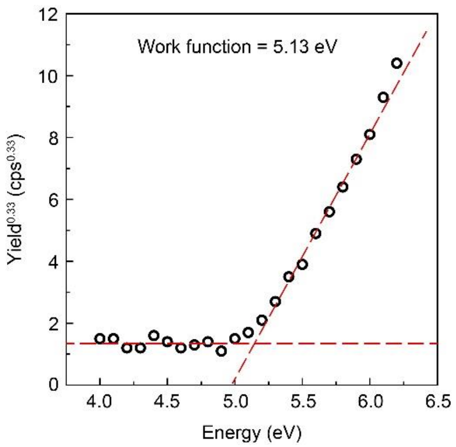


**Supplementary Fig. 27.** Work function of conductive PEDOT:PSS with 5 mg mL$^{-1}$ ION E additive.

**Supplementary Table 4.** Initial photovoltaic parameters of the IS-OPVs measured under 100 mW $cm^{-2}$ AM 1.5G illumination

| | $J_{SC}$ (mA $cm^{-2}$) | $V_{OC}$ (V) | FF | PCE (%) |
|---|---|---|---|---|
| **PM6:Y6** | 25.87 ± 0.06 | 0.78 ± 0.00 | 0.65 ± 0.01 | 13.14 ± 0.06 |
| **Ter-D18:Y6** | 25.19 ± 0.16 | 0.84 ± 0.00 | 0.67 ± 0.00 | 14.18 ± 0.04 |

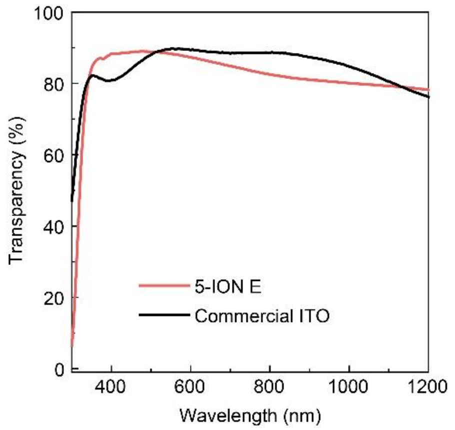


**Supplementary Fig. 28.** Transparency of conductive PEDOT:PSS films with 5-ION E and commercial ITO.

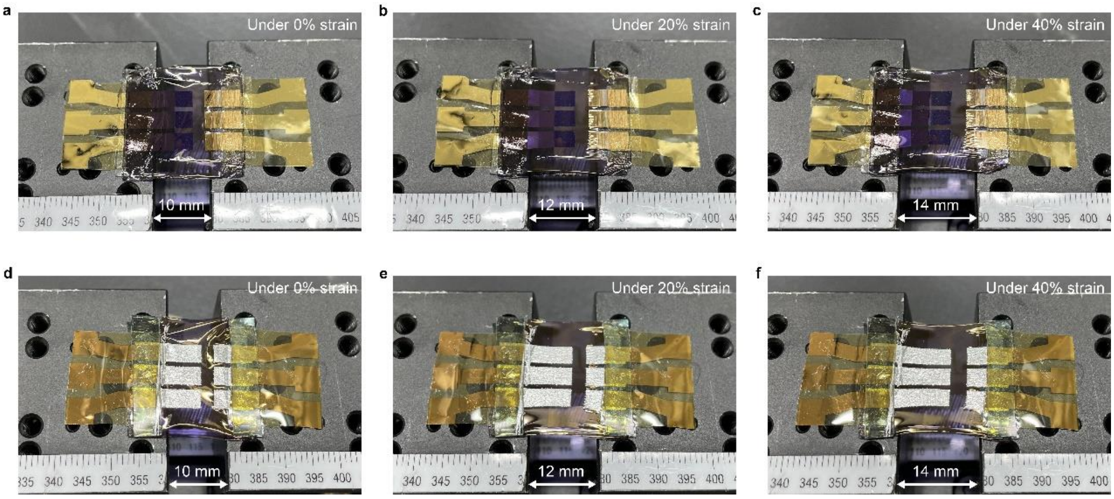


**Supplementary Fig. 29.** Photographs of a–c) the back side and d–f) the front side of an IS-OPV device subjected to different tensile strains.

**Supplementary Table 5.** Photovoltaic parameters of IS-OPVs as a function of strain measured under 100 mW cm$^{-2}$ AM 1.5G illumination.

| Active system | Strain (%) | $J_{SC}$ (mA cm$^{-2}$) | $V_{OC}$ (V) | FF | PCE (%) |
|---|---|---|---|---|---|
| PM6:Y6 | 0 | 25.87 ± 0.06 | 0.78 ± 0.00 | 0.65 ± 0.01 | 13.14 ± 0.06 |
| | 10 | 25.49 ± 0.32 | 0.78 ± 0.00 | 0.64 ± 0.00 | 12.73 ± 0.13 |
| | 20 | 24.4 ± 0.15 | 0.78 ± 0.00 | 0.63 ± 0.01 | 12.33 ± 0.27 |
| | 30 | 22.83 ± 0.36 | 0.76 ± 0.02 | 0.63 ± 0.01 | 10.94 ± 0.29 |
| | 40 | 19.92 ± 0.24 | 0.75 ± 0.00 | 0.57 ± 0.01 | 8.53 ± 0.23 |
| | 50 | 15.29 ± 0.68 | 0.67 ± 0.01 | 0.47 ± 0.04 | 4.85 ± 0.23 |
| Ter-D18:Y6 | 0 | 25.19 ± 0.16 | 0.84 ± 0.00 | 0.67 ± 0.00 | 14.18 ± 0.04 |
| | 10 | 25.02 ± 0.17 | 0.84 ± 0.00 | 0.67 ± 0.01 | 14.06 ± 0.20 |
| | 20 | 24.34 ± 0.21 | 0.84 ± 0.00 | 0.67 ± 0.00 | 13.69 ± 0.23 |
| | 30 | 24.11 ± 0.28 | 0.83 ± 0.00 | 0.66 ± 0.00 | 13.20 ± 0.18 |
| | 40 | 23.47 ± 0.29 | 0.82 ± 0.00 | 0.64 ± 0.00 | 12.42 ± 0.22 |
| | 50 | 22.71 ± 0.35 | 0.82 ± 0.00 | 0.62 ± 0.01 | 11.57 ± 0.18 |
| | 60 | 22.3 ± 0.38 | 0.80 ± 0.01 | 0.59 ± 0.00 | 10.63 ± 0.21 |
| | 70 | 20.74 ± 0.56 | 0.75 ± 0.02 | 0.55 ± 0.02 | 8.49 ± 0.37 |
| | 80 | 16.84 ± 0.87 | 0.67 ± 0.04 | 0.44 ± 0.07 | 4.97 ± 0.59 |

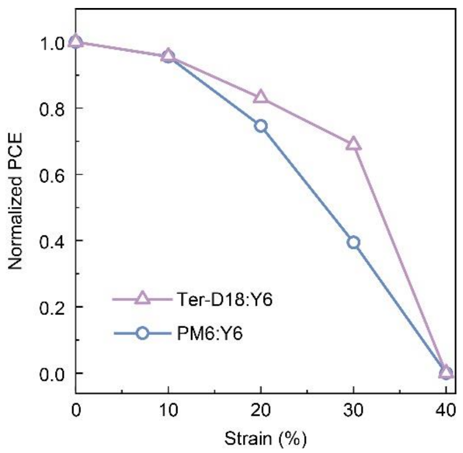


**Supplementary Fig. 30.** Normalised PCEs of the IS-OPVs using 0-ION E conductive PEDOT:PSS electrodes under tensile strain.

**Supplementary Table 6.** Photovoltaic parameters of devices using 0-ION E conductive PEDOT:PSS electrodes as a function of strain measured under 100 mW cm$^{-2}$ AM 1.5G illumination.

| Active system | Strain (%) | $J_{SC}$ (mA cm$^{-2}$) | $V_{OC}$ (V) | FF | PCE (%) |
|---|---|---|---|---|---|
| PM6:Y6 | 0 | 25.36 | 0.79 | 0.65 | 13.07 |
| | 10 | 25.04 | 0.79 | 0.64 | 12.49 |
| | 20 | 22.30 | 0.77 | 0.57 | 9.76 |
| | 30 | 17.37 | 0.67 | 0.45 | 5.16 |
| | 40 | 0.00 | 0.16 | 0.26 | 0.00 |
| Ter-D18:Y6 | 0 | 24.95 | 0.83 | 0.67 | 13.92 |
| | 10 | 24.74 | 0.83 | 0.65 | 13.32 |
| | 20 | 23.35 | 0.81 | 0.61 | 11.57 |
| | 30 | 21.52 | 0.77 | 0.58 | 9.60 |
| | 40 | 0.00 | 0.25 | 0.26 | 0.00 |

**Supplementary Table 7.** Device structures and mechanical and photovoltaic performance data of recently reported IS-OPVs. The $PCE_{80\%}$ values were estimated by interpolating the data provided in the respective papers.

| Device structure | Active layer | Initial PCE (%) | Strain at $PCE_{80\%}$ (%) | Ref. |
|---|---|---|---|---|
| TPU/AgNW/PEDOT:PSS/active layer/EGaIn | PTB7-Th:IEICO-4F | 10.1 | 12 | [14] |
| | | 8.6 | 15.2 | |
| | | 7.5 | 28.3 | |
| TPU/PH1000/AI4083/active layer/PNDIT-F3N-Br/EGaIn | PM6:Y7 | 11.2 | 12.4 | [15] |
| | PM6:PCBM | 5.7 | 5.1 | |
| | PCE12:N2200 | 5 | 42.3 | |
| TPU/PH1000/AI4083/active layer/PNDIT-F3N-Br/EGaIn | PM6:Y7:N2200 | 11.71 | 19.9 | [16] |
| TPU/PH1000/AI4083/active layer/PNDIT-F3N-Br/EGaIn | PhAm5:Y7 | 12.76 | 31.6 | [17] |
| TPU/PH1000/AI4083/active layer/PNDIT-F3N-Br/EGaIn | PM6-OEG5:BTP-eC9 | 12.05 | 22 | [18] |
| TPU/GL:PH1000/AI4083/active layer/PNDIT-F3N-Br/EGaIn | PM6:Y6:5wt% BAC | 13.4 | 20 | [19] |
| TPU/PH1000/AI4083/active layer/PNDIT-F3N-Br/EGaIn | PBDB-T:PYFS-Reg | 10.64 | 36.7 | [20] |
| TPU-AgNWs/PEDOT:PSS/active layer/Azo-AgNWs-AZO | PM6:BTP-eC9 | 10.9 | 9.9 | [21] |
| TPU/modified PH1000/ AI4083/active layer/ PNDIT-F3N-Br/EGaIn | PM7-Thy10:L8-BO | 13.69 | 43.1 | [22] |
| TPU/modified PH1000/ AI4083/active layer/ PNDIT-F3N-Br/EGaIn | PM6:Y6-BO:N2200 | 10.2 | 40 | [23] |
| **PU//conductive PH1000//AI4083//active layer//EGaIn** | **Ter-D18:Y6** | **14.18** | **52** | **Present study** |
| | **PM6:Y6** | **13.14** | **32** | |

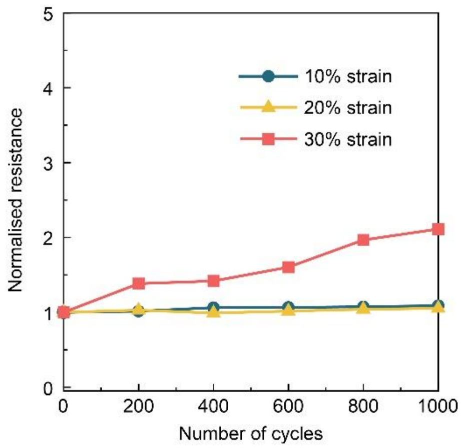


**Supplementary Fig. 31.** Normalised resistance of PU//5 mg $mL^{-1}$ ION E-incorporated conductive PEDOT:PSS film under repetitive stretch–release cycles at 10%, 20%, and 30% tensile strains.

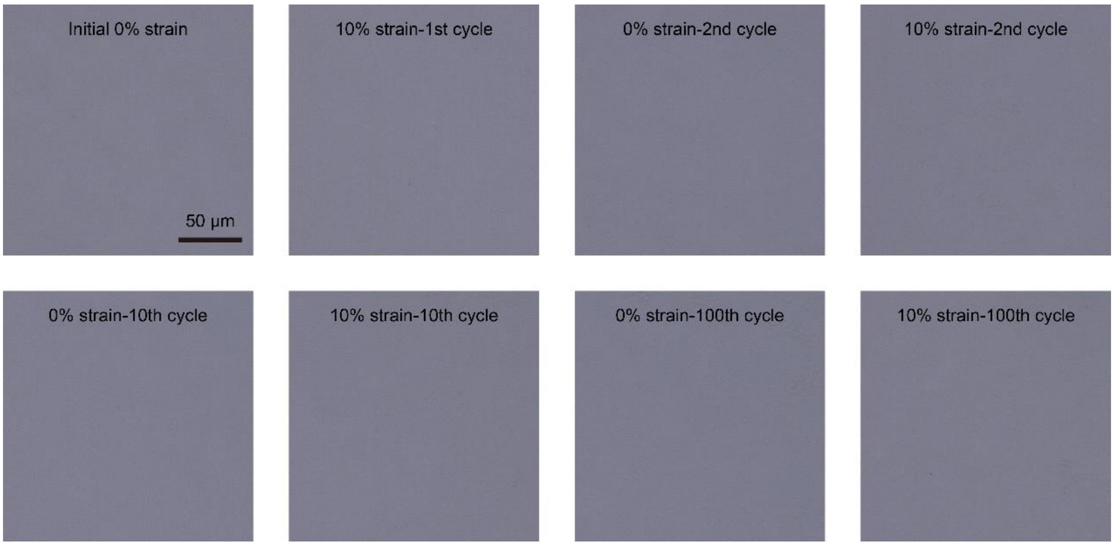


**Supplementary Fig. 32.** OM images of released and stretched freestanding PU//5 mg $mL^{-1}$ ION E-incorporated conductive PEDOT:PSS films at the 1st, 2nd, 10th, and 100th stretch–release cycles under 10% strain.

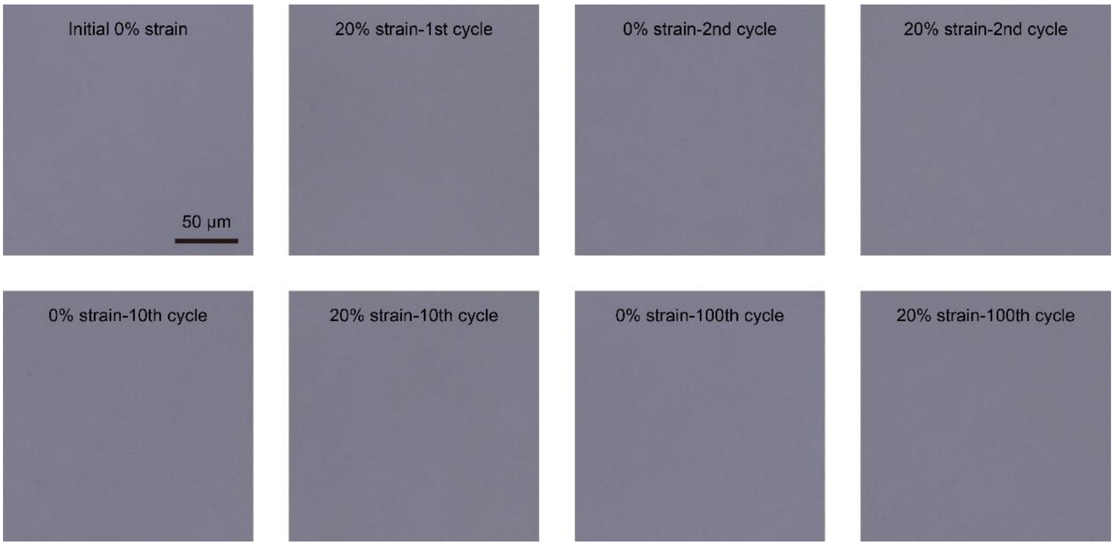


**Supplementary Fig. 33.** OM images of released and stretched freestanding PU//5 mg $mL^{-1}$ ION E-incorporated conductive PEDOT:PSS films at the 1st, 2nd, 10th, and 100th stretch–release cycles under 20% strain.

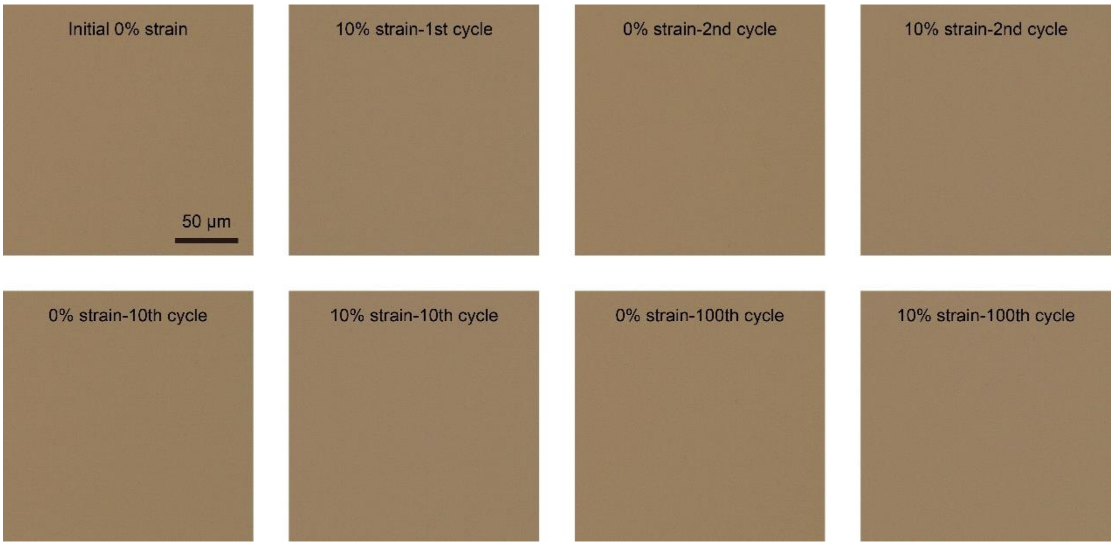


**Supplementary Fig. 34.** OM images of released and stretched freestanding composite films with 5 mg $mL^{-1}$ ION E additive and Ter-D18:Y6 active system at the 1st, 2nd, 10th, and 100th stretch–release cycles under 10% strain.

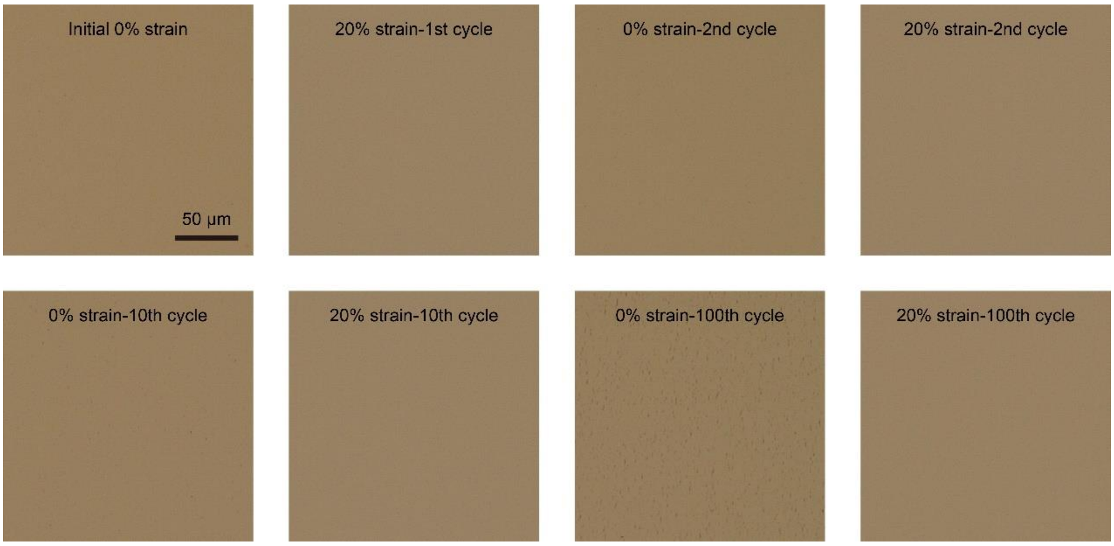

**Supplementary Fig. 35.** OM images of released and stretched freestanding composite films with 5 mg mL$^{-1}$ ION E additive and Ter-D18:Y6 active system at the 1st, 2nd, 10th, and 100th stretching–release cycles under 20% strain.

**Supplementary Note 7. Surface Morphology of PU//conductive PEDOT:PSS Films with 5-ION E and Composite Films with Ter-D18:Y6 Active Layer under Cyclic Stretching at 10% and 20% Strains for 100 Cycles**

No visible features were observed in the PU//conductive PEDOT:PSS films during strain–release cycles at different tensile strains, suggesting that the films exhibited decent cycling durability, owing to the improved stretchability and strengthened interfacial adhesion (Supplementary Figs. 19, 20).[24] Accordingly, the composite films also exhibited remarkable mechanical durability by redistributing the strain to the underlying layers. Specifically, the uppermost active layer showed a flat surface after 100 stretching cycles at 10% strain (Supplementary Fig. 21), and only sparse crumples appeared after 100 stretching cycles at 20% strain (Supplementary Fig. 22). Note that these point defects were not cracks because they maintained perfect surface integrity under stretched conditions.

**Supplementary Table 8.** Device structures and mechanical durability data of recently reported IS-OPVs.

| Device structure | Number of cycles | Strain | Post-cycling PCE (% of the initial value) | Ref. |
|---|---|---|---|---|
| *PU/conductive PH1000/AI4083/Ter-D18:Y6/EGaIn* | *100* | *10%* | *95%* | *Present study* |
| | | *20%* | *85%* | |
| TPU/modified PH1000/ AI4083/ PM6:Y6-BO:N2200/ PNDIT-F3N-Br/EGaIn | 1000 | 10% | 90.4% | 23 |
| TPU/PH1000/AI4083/ PM6:Y7/PNDIT-F3N-Br/EGaIn | 1000 | 10% | 74% | 15 |
| TPU/PH1000/AI4083/ PhAm5:Y7/PNDIT-F3N-Br/EGaIn | 120 | 15% | 86% | 17 |
| TPU/AgNW/PEDOT:PSS/PTB7-Th:IEICO-4F/EGaIn | 300 | 20% | 47% | 14 |
| TPU/PH1000/AI4083/PM6:Y7:N2200/PNDIT-F3N-Br/EGaIn | 100 | 15% | 84% | 16 |
| TPU/GL:PH1000/AI4083/PM6:Y6:5wt% BAC/PNDIT-F3N-Br/EGaIn | 100 | 20% | 81% | 19 |
| TPU/PH1000/AI4083/ PM6-OEG5:BTP-eC9/PNDIT-F3N-Br/EGaIn | 100 | 10% | 86% | 18 |

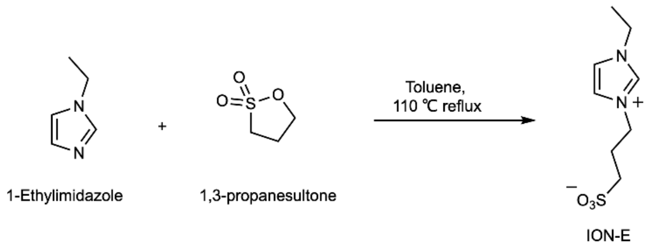


**Supplementary Fig. 36.** Synthesis of ION E.

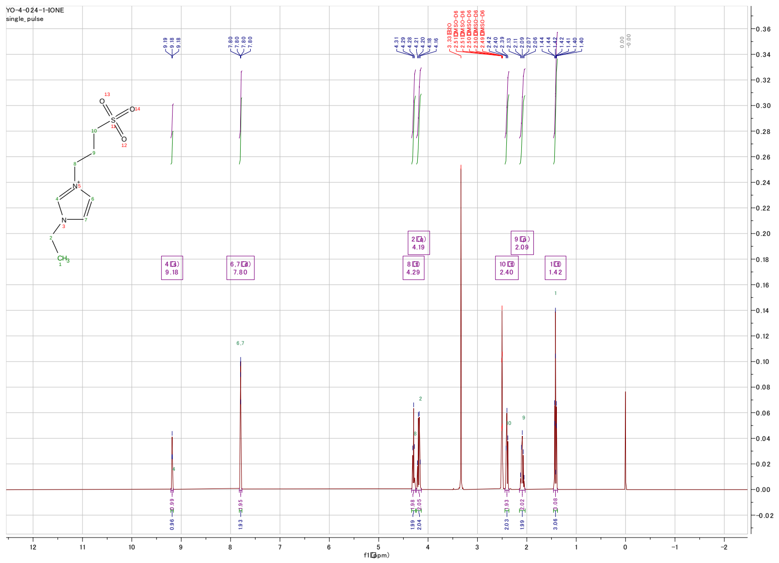


**Supplementary Fig. 37.** $^1$H NMR spectrum of ION E.

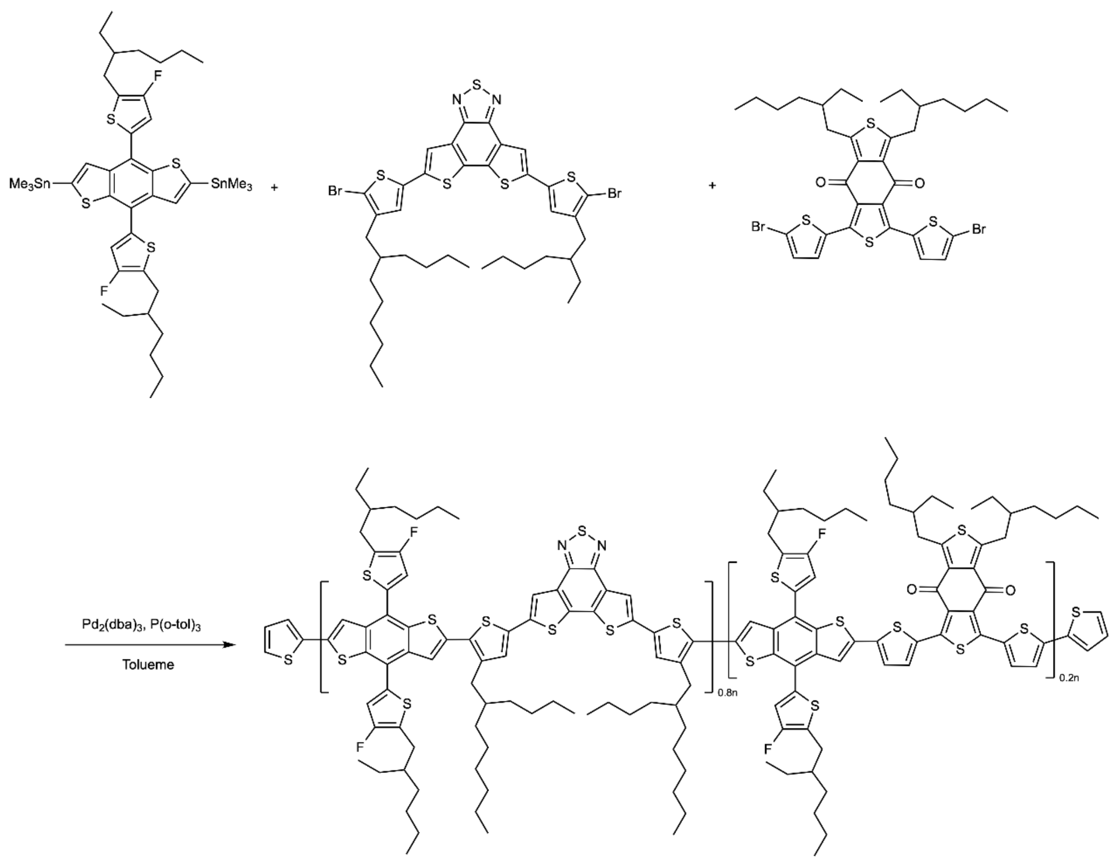


**Supplementary Fig. 38.** Synthesis of Ter-D18.

## Supplementary References


1. Zalesskiy, S.S. & Ananikov, V.P. Pd2(dba)3 as a Precursor of Soluble Metal Complexes and Nanoparticles: Determination of Palladium Active Species for Catalysis and Synthesis. Organometallics **31**, 2302-2309 (2012).
2. Tahk, D., Lee, H.H. & Khang, D.-Y. Elastic Moduli of Organic Electronic Materials by the Buckling Method. Macromol. **42**, 7079-7083 (2009).
3. Greczynski, G. et al. Photoelectron spectroscopy of thin films of PEDOT–PSS conjugated polymer blend: a mini-review and some new results. J. Electron Spectros. Relat. Phenomena **121**, 1-17 (2001).
4. Liu, T. et al. Low-Work-Function PEDOT Formula as a Stable Interlayer and Cathode for Organic Solar Cells. Adv. Funct. Mater. **31**, 2107250 (2021).
5. Khong, S.H. et al. General Photo-Patterning of Polyelectrolyte Thin Films via Efficient Ionic Bis(Fluorinated Phenyl Azide) Photo-Crosslinkers and their Post-Deposition Modification. Adv. Funct. Mater. **17**, 2490-2499 (2007).
6. He, H. et al. Biocompatible Conductive Polymers with High Conductivity and High Stretchability. ACS Appl. Mater. Interfaces **11**, 26185-26193 (2019).
7. He, H. et al. Salt-induced ductilization and strain-insensitive resistance of an intrinsically conducting polymer. Sci. Adv. **8**, eabq8160.
8. Brus, V.V., Proctor, C.M., Ran, N.A. & Nguyen, T.-Q. Capacitance Spectroscopy for Quantifying Recombination Losses in Nonfullerene Small-Molecule Bulk Heterojunction Solar Cells. Adv. Energy Mater. **6**, 1502250 (2016).
9. Steele, J.A. et al. How to GIWAXS: Grazing Incidence Wide Angle X-Ray Scattering Applied to Metal Halide Perovskite Thin Films. Adv. Energy Mater. **13**, 2300760 (2023).
10. Mahmood, A. & Wang, J.-L. A Review of Grazing Incidence Small- and Wide-Angle X-Ray Scattering Techniques for Exploring the Film Morphology of Organic Solar Cells. Solar RRL **4**, 2000337 (2020).
11. Zhang, M., Guo, X., Ma, W., Ade, H. & Hou, J. A Large-Bandgap Conjugated Polymer for Versatile Photovoltaic Applications with High Performance. Adv. Mater. **27**, 4655-4660 (2015).
12. He, K., Chen, N., Wang, C., Wei, L. & Chen, J. Method for Determining Crystal Grain Size by X-Ray Diffraction. Cryst. Res. Technol. **53**, 1700157 (2018).
13. Lu, H. et al. Random Terpolymer Enabling High-Efficiency Organic Solar Cells Processed by Nonhalogenated Solvent with a Low Nonradiative Energy Loss. Adv. Funct. Mater. **32**, 2203193 (2022).
14. Wang, Z. et al. Intrinsically Stretchable Organic Solar Cells beyond 10% Power Conversion Efficiency Enabled by Transfer Printing Method. Adv. Funct. Mater. **31**, 2103534 (2021).
15. Noh, J. et al. Intrinsically Stretchable Organic Solar Cells with Efficiencies of over 11%. ACS Energy Lett. **6**, 2512-2518 (2021).
16. Lee, J.-W. et al. Intrinsically-Stretchable, Efficient Organic Solar Cells Achieved by High-Molecular-Weight, Electro-Active Polymer Acceptor Additives. Adv. Energy Mater. **12**, 2200887 (2022).

17. Lee, J.-W. et al. Intrinsically Stretchable, Highly Efficient Organic Solar Cells Enabled by Polymer Donors Featuring Hydrogen-Bonding Spacers. Adv. Mater. **34**, 2207544 (2022).
18. Lee, J.-W. et al. Intrinsically Stretchable and Non-Halogenated Solvent Processed Polymer Solar Cells Enabled by Hydrophilic Spacer-Incorporated Polymers. Adv. Energy Mater. **12**, 2202224 (2022).
19. Wang, Z. et al. Intrinsically Stretchable Organic Solar Cells with Simultaneously Improved Mechanical Robustness and Morphological Stability Enabled by a Universal Crosslinking Strategy. Small **18**, 2201589 (2022).
20. Lee, J.-W. et al. Sequentially regular polymer acceptors featuring flexible spacers for high-performance and mechanically robust all-polymer solar cells. Energy Environ. Sci. **15**, 4672-4685 (2022).
21. Huang, J. et al. Intrinsically stretchable, semi-transparent organic photovoltaics with high efficiency and mechanical robustness via a full-solution process. Energy Environ. Sci. **16**, 1251-1263 (2023).
22. Wan, Q. et al. High-Performance Intrinsically Stretchable Polymer Solar Cell with Record Efficiency and Stretchability Enabled by Thymine-Functionalized Terpolymer. J. Am. Chem. Soc. **145**, 11914-11920 (2023).
23. Lee, S. et al. Intrinsically Stretchable Organic Solar Cells without Cracks under 40% Strain. Adv. Energy Mater., 2300533 (2023).
24. Kang, J. et al. Tough-interface-enabled stretchable electronics using non-stretchable polymer semiconductors and conductors. Nat. Nanotechnol. **17**, 1265-1271 (2022).
