# Supplementary material for: Intrinsically stretchable organic photovoltaics by redistributing strain to PEDOT:PSS with enhanced stretchability and interfacial adhesion
Source: Nat Commun. 2024 Jun 8;15:4902. doi: 10.1038/s41467-024-49352-4 (PMC11162488; doi:10.1038/s41467-024-49352-4)
Supplement: Supplementary file 3 — Reporting Summary [file 41467_2024_49352_MOESM3_ESM.pdf]

Corresponding author(s): Kenjiro Fukuda, Takao Someya

Solar Cells Reporting Summary

Nature Portfolio wishes to improve the reproducibility of the work that we publish. This form is intended for publication with all accepted papers reporting the characterization of photovoltaic devices and provides structure for consistency and transparency in reporting. Some list items might not apply to an individual manuscript, but all fields must be completed for clarity.

For further information on Nature Research policies, including our data availability policy, see Authors & Referees.

Experimental design

Please check the following details are reported in the manuscript, and provide a brief description or explanation where applicable.

1. Dimensions

Item	Yes/No	Description
Area of the tested solar cells	☒ Yes ☐ No	The active area is 4 mm^2. Described in "Results and Discussion" section. / *Explain why this information is not reported/not relevant.*
Method used to determine the device area	☒ Yes ☐ No	The active area is defined by a photo mask with an open window of 2 mm × 2 mm. Described in "Results and Discussion" section. / *Explain why this information is not reported/not relevant.*

2. Current-voltage characterization

Item	Yes/No	Description
Current density-voltage (J-V) plots in both forward and backward direction	☐ Yes ☒ No	J-V plot only in forward direction.
Voltage scan conditions	☒ Yes ☐ No	The solar cells are measured in forward direction. / *Explain why this information is not reported/not relevant.*
Test environment	☒ Yes ☐ No	The devices were characterized in an ambient atmosphere. Described in "Experimental Section" section. / *Explain why this information is not reported/not relevant.*
Protocol for preconditioning of the device before its characterization	☐ Yes ☒ No	*Provide a description of the protocol.* / No preconditioning of the device before its characterization.
Stability of the J-V characteristic	☐ Yes ☒ No	*Provide a description of the method used. The stability of the J-V characteristic can be verified with time evolution of the maximum power point or with the photocurrent at maximum power point; see ref. 5 for details.* / We only measured the performance change under various tensile strains

3. Hysteresis or any other unusual behaviour

Item	Yes/No	Description
Description of the unusual behaviour observed during the characterization	☐ Yes ☒ No	*Provide a description of hysteresis or any other unusual behaviour observed during the characterization.* / No hysteresis or other unusual behavior was observed during the characterization.
Related experimental data	☐ Yes ☒ No	*Provide a description of the related experimental data.* / N/A

4. Efficiency

Item	Yes/No	Description
External quantum efficiency (EQE) or incident photons to current efficiency (IPCE)	☒ Yes ☐ No	EQE spectra of cells are shown in supplementary Fig. 16d and Fig. 5c. / *Explain why this information is not reported/not relevant.*
A comparison between the integrated response under the standard reference spectrum and the response measure under the simulator	☒ Yes ☐ No	Integrated EQE under AM1.5 is comparable to current density under the 1-sun simulator illumination. / *Explain why this information is not reported/not relevant.*

April 2023

For tandem solar cells, the bias illumination and bias voltage used for each subcell

☐ Yes — *Provide a description of the measurement conditions.*
☒ No — No tandem cells are reported in the work.

5. Calibration

Light source and reference cell or sensor used for the characterization

☒ Yes — Described in "Methods" section. The light source is XES-40S3, SAN-EI ELECTRIC.
☐ No — *Explain why this information is not reported/not relevant.*

Confirmation that the reference cell was calibrated and certified

☒ Yes — Described in "Methods" section. The reference diode is BS-520BK Bunkoukeiki.
☐ No — *Explain why this information is not reported/not relevant.*

Calculation of spectral mismatch between the reference cell and the devices under test

☐ Yes — *Provide a value of the spectral mismatch and/or a description of how it has been taken into account in the measurements.*
☒ No — Mismatch is not calculated.

6. Mask/aperture

Size of the mask/aperture used during testing

☒ Yes — The size of the mask is 2 mm × 2 mm.
☐ No — *Explain why this information is not reported/not relevant.*

Variation of the measured short-circuit current density with the mask/aperture area

☐ Yes — *Report the difference in the short-circuit current density values measured with the mask and aperture area.*
☒ No — We used the same photo mask for all the measurements.

7. Performance certification

Identity of the independent certification laboratory that confirmed the photovoltaic performance

☐ Yes — *Identify the independent certification laboratory.*
☒ No — This work mainly focuses on the intrinsic stretchability of the devices. We have not certified the efficiency by the independent laboratory.

A copy of any certificate(s)

☐ Yes — *Certificate copies should be provided in the Supplementary information. Please state the supplementary item number.*
☒ No — No certification.

8. Statistics

Number of solar cells tested

☐ Yes — *Report how many solar cells have been tested, specifying the number of individual substrates.*
☒ No — we show the average values and their standard deviation.

Statistical analysis of the device performance

☐ Yes — *State where this information can be found in the text.*
☒ No — we show the average values and their standard deviation.

9. Long-term stability analysis

Type of analysis, bias conditions and environmental conditions

☐ Yes — *Provide a description of the type of analysis, bias conditions and environmental conditions (e.g. illumination type, temperature, atmosphere humidity, encapsulation method, preconditioning temperature, bias) for each long-term stability analysis carried out; see ref. 7 and 8 for details.*
☒ No — We mainly focus on the stretchability and mechanical durability of the devices.
